# Neutrophil-to-Lymphocyte Ratio as a Biomarker Predicting Overall Survival after Chemoembolization for Intermediate-Stage Hepatocellular Carcinoma

**DOI:** 10.3390/cancers13112830

**Published:** 2021-06-06

**Authors:** Hee Ho Chu, Jin Hyoung Kim, Ju Hyun Shim, Dong Il Gwon, Heung-Kyu Ko, Ji Hoon Shin, Gi-Young Ko, Hyun-Ki Yoon, Nayoung Kim

**Affiliations:** 1 Asan Medical Center, Department of Radiology, University of Ulsan College of Medicine, 88 Olympic-ro 43-gil, Songpa-gu, Seoul 05505, Korea; angiochu@amc.seoul.kr (H.H.C.); radgwon@amc.seoul.kr (D.I.G.); hk.ko@amc.seoul.kr (H.-K.K.); jhshin@amc.seoul.kr (J.H.S.); kogy@amc.seoul.kr (G.-Y.K.); hkyoon@amc.seoul.kr (H.-K.Y.); 2 Liver Center, Asan Medical Center, Department of Gastroenterology, University of Ulsan College of Medicine, Seoul 05505, Korea; s5854@amc.seoul.kr; 3 Asan Medical Center, Department of Clinical Epidemiology and Biostatistics, University of Ulsan College of Medicine, 88, Olympic-Ro 43-Gil, Songpa-Gu, Seoul 05505, Korea; nyny0803@amc.seoul.kr

**Keywords:** hepatocellular carcinoma, intermediate-stage, transarterial chemoembolization, neutrophil-to-lymphocyte ratio

## Abstract

**Simple Summary:**

Baseline neutrophil-to-lymphocyte ratio ≥3 was a robust independent predictor of overall survival after chemoembolization for intermediate-stage hepatocellular carcinoma, with the predictive value verified by cross-validation.

**Abstract:**

The clinical impact of neutrophil-to-lymphocyte ratio (NLR) in predicting outcomes in hepatocellular carcinoma (HCC) patients treated with transarterial chemoembolization (TACE) remain unclear, and additional large-scale studies are required. This retrospective study evaluated outcomes in treatment-naïve patients who received TACE as first-line treatment for intermediate-stage HCC between 2008 and 2017. Patients who underwent TACE before and after 2013 were assigned to the development (*n* = 495) and validation (*n* = 436) cohorts, respectively. Multivariable Cox analysis identified six factors predictive of outcome, including NLR, which were used to create models predictive of overall survival (OS) in the development cohort. Risk scores of 0–3, 4–7, and 8–12 were defined as low, intermediate, and high risk, respectively. Median OS times in the low-, medium-, and high-risk groups in the validation cohort were 48.1, 24.3, and 9.7 months, respectively (*p* < 0.001). Application to the validation cohort of time-dependent ROC curves for models predictive of OS showed AUC values of 0.72 and 0.70 at 3 and 5 years, respectively. Multivariable logistic regression analysis found that NLR ≥ 3 was a significant predictor (odds ratio, 3.4; *p* < 0.001) of disease progression 6 months after TACE. Higher baseline NLR was predictive of poor prognosis in patients who underwent TACE for intermediate-stage HCC.

## 1. Introduction

Many patients with hepatocellular carcinoma (HCC) are diagnosed with intermediate-to-advanced stage disease, where curative treatment is not feasible [1]. Overall survival (OS) of these patients can vary between 6 and 20 months [2]. Transarterial chemoembolization (TACE) is a well-established treatment option for patients with unresectable HCC, with randomized controlled trials showing that survival is improved after TACE [2]. Factors prognostic of OS in patients with HCC undergoing TACE include tumor size, tumor multiplicity, vascular invasion, extrahepatic spread, underlying liver functional reserve, α-fetoprotein (AFP) concentration, and performance status [3,4,5,6]. Nevertheless, precise prediction of outcomes remains challenging due to the many variables in patients undergoing TACE for unresectable HCC [3,4,5,6].

The prognostic value of systemic immune markers, such as neutrophil-to-lymphocyte ratio (NLR), has been investigated in various types of cancer [7,8]. Neutrophils facilitate carcinogenesis and angiogenesis, and promote the motility of cancer cells, thereby enhancing tumor invasion and metastasis [9]. Lymphocyte depletion reflects an impaired antitumor response, and lymphopenia is associated with poor outcomes in cancer patients [10]. NLR may predict outcomes following treatment of HCC, with several studies reporting that a higher NLR predicted HCC recurrence and was associated with poorer survival following various treatment modalities in patients with HCC [11,12,13]. Moreover, higher pretreatment NLR was shown to be associated with poor outcomes in HCC patients undergoing TACE [13]. Despite these findings, however, studies assessing the clinical impact of NLR in HCC patients undergoing TACE have yielded inconsistent results, suggesting the need for additional large-scale studies [13]. The present study therefore evaluated the prognostic value of NLR as a predictor of outcomes after TACE in a cohort of patients with intermediate-stage HCC (Barcelona Clinical Liver Cancer (BCLC) stage B), conditions considered optimal indications for TACE.

## 2. Materials and Methods

### 2.1. Study Design and Patients

Data from treatment-naïve patients who received TACE as first-line treatment for intermediate-stage (BCLC B) HCC [14] between January 2008 and December 2017 were retrospectively evaluated. Patients were excluded if they had undergone TACE for preoperative purposes, if they had undergone liver transplantation or surgical resection after TACE, if they were lost to follow-up, or if they had a previous or current malignancy other than HCC. Patients who underwent TACE before and after 2013 were assigned to the development and validation cohorts, respectively.

The study design was approved by the Institutional Review Board of our institution, which waived the requirement for patient informed consent because of the retrospective, anonymized nature of the study.

### 2.2. Transarterial Chemoembolization

Details of the TACE procedure have been described previously [15]. Briefly, TACE was performed by one of six highly experienced interventional radiologists, each with at least 10 years of experience. Using a 1.8-2.4-F microcatheter (Renegade; Boston Scientific, Cork, Ireland, Progreat; Terumo, Japan, Carnelian; Tokai, Japan), a 1:1 emulsion of Lipiodol (Guerbet, Roissy, France; maximum dose, 20 mL) and cisplatin (2 mg/kg) was infused selectively into a segmental, subsegmental, or more peripheral-level feeding artery, followed by infusion of Gelfoam particles (Upjohn, Kalamazoo, MI, USA) until sufficient stasis of arterial flow. Care was taken to avoid non-target embolization of the normal liver parenchyma. All HCCs were embolized in a single TACE session. Patients underwent repeat TACE when follow-up CT or MRI scans detected residual tumor, tumor growth, or new tumors, as long as the patient’s underlying liver function and general condition could tolerate TACE.

### 2.3. Study End Point

The primary study end point was to detect a significant relationship between NLR and OS, after adjusting for other potential variables, including age, sex, serum AFP concentration (≥200 mg/dL vs. <200 mg/dL), Child–Pugh classification (A vs. B), tumor type (infiltrative vs. nodular), maximal tumor size (>5 cm vs. ≤5 cm), and tumor number (≥4 vs. <4) [16,17]. Serum complete blood count levels just before TACE were utilized to calculate NLR; by dividing the absolute neutrophil count by absolute lymphocyte count.

NLR was dichotomized as ≥3 and <3, as previously described [18], and evaluated as a significant indicator of OS. Cross-validation was performed to increase the generalizability and stability of the study results. That is, the pretreatment risk prediction model derived from the development cohort was applied to a separate validation cohort.

A secondary study end point was to detect a significant relationship between NLR and 6 month tumor response after TACE, as evaluated by dynamic CT or MRI scans and after adjustment for other potential variables. Tumor response was evaluated using the mRECIST criteria, and categorized as complete response (CR), partial response (PR), stable disease (SD), and progressive disease (PD) [19].

### 2.4. Statistical Analysis

Cumulative survival curves were generated using the Kaplan–Meier method and compared using log-rank tests. OS was measured in months from the time of the initial TACE session to patient death from any cause. Patients who were alive at the end of this study (November 2020) were censored for the survival rate calculations.

To generate the pretreatment risk prediction model for OS, a multivariable Cox proportional hazards model using the backward elimination method was used in the development cohort. Variables with *p* < 0.05 on univariable analyses were included in the multivariable analyses. Risk points were assigned to variables with *p* < 0.05 on multivariable analysis of the developing cohort. The β regression coefficient of each variable was used to calculate risk points [20]. This point algorithm was tested in the validation cohort, with risk scores determined as the sum of these points for the corresponding predictors. Patients in the development and validation cohorts were classified into three groups according to risk scores, with OS curves generated using the Kaplan–Meier method. Time-dependent receiver operating characteristic (ROC) curves were utilized to analyze the performance of the pretreatment risk prediction model in the development and validation cohorts [21].

To identify the factors associated with PD 6 month after TACE, variables with *p* < 0.05 on univariable analyses were entered into multivariable logistic regression analyses. Statistical analyses were performed using SPSS (version 21.0; SPSS, Chicago, IL, USA) and R (version 3.6.1; R Development Core Team, Auckland, New Zealand) software. Two-sided *p-*values < 0.05 were considered statistically significant.

## 3. Results

### 3.1. Patient Characteristics

**Figure 1:**
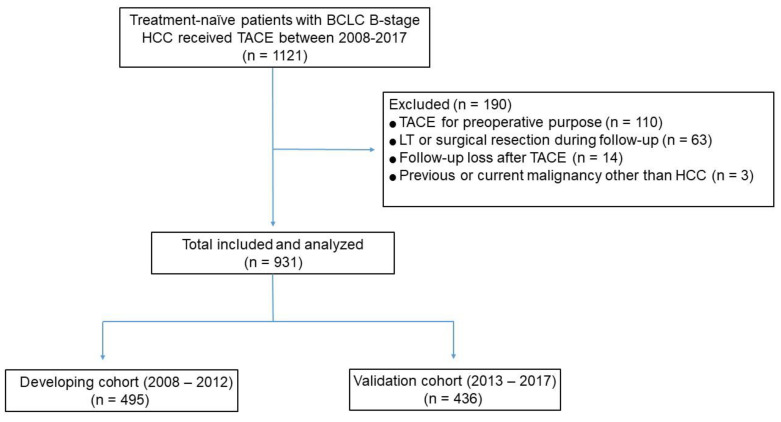
Flow diagram of the study population.

Of the 1121 consecutive patients who received TACE as first-line treatment for intermediate-stage HCC, 931 were included, 495 in the development cohort, and 436 in the validation cohort (Figure 1). Except for age (*p* < 0.001), baseline demographic and clinical characteristics were similar in the development and validation cohorts (Table 1). The median largest tumor size in all 931 study patients was 5.3 cm (interquartile range (IQR), 3.8–8.3 cm).

### 3.2. Model Predicting Overall Survival

Patients were followed up for a median 31 months (IQR, 16.3–52.2 months), during which time 723 (77.6%) patients died, and 208 (22.4%) remained alive. The median OS of all 931 patients was 31.1 months (95% confidence interval (CI), 28.5–33.7 months). The OS rates of the whole cohort at 1, 3, 5, and 10 years were 81.7%, 45%, 28.2%, and 14.6%, respectively.

Multivariable Cox regression analyses of the development cohort showed that tumor diameter > 5 cm (hazard ratio (HR), 1.30; 95% CI, 1.06–1.61; *p* = 0.013), tumor number ≥ 4 (HR, 1.67; 95% CI, 1.35–2.05; *p* < 0.001), infiltrative tumor type (HR, 2.28; 95% CI, 1.73–3.01; *p* < 0.001), AFP ≥ 200 ng/mL (HR, 1.23; 95% CI, 1.01–1.50; *p* = 0.042), NLR ≥ 3 (HR, 1.41; 95% CI, 1.10–1.81; *p* = 0.007), and Child–Pugh B (HR, 1.66; 95% CI, 1.24–2.23; *p* < 0.001) were significantly associated with OS rate after TACE (Table 2).

**Figure 2:**
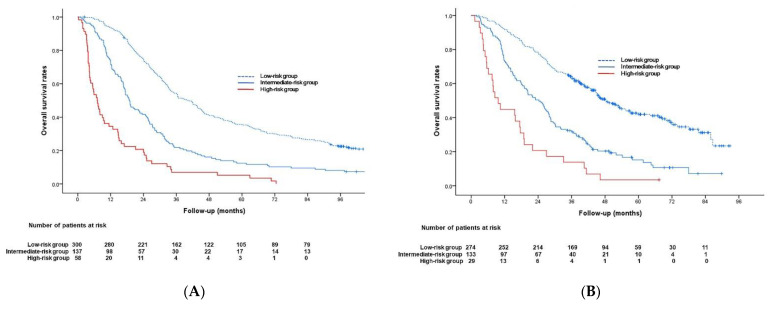
Kaplan–Meier analysis of overall survival in the (**A**) development and (**B**) validation cohorts.

Based on the results of multivariable Cox analyses in the development cohort, a pretreatment risk prediction model was generated using six predictive factors. The β regression coefficients of these six factors and their corresponding rounded risk points in the development cohort are shown in Table 2. Risk scores for all patients in the validation cohort were calculated as the sum of these corresponding risk points, and patients with scores of 0–3 (*n* = 274), 4–7 (*n* = 133), and 8–12 (*n* = 29) were classified into those at low, intermediate, and high risk, respectively. The median OS times in the low-, intermediate-, and high-risk groups were 40.8 months (95% CI, 35.2–46.4 months), 18.9 months (95% CI, 16.2–21.6 months), and 7.1 months (95% CI, 4.9–9.3 months), respectively, in the development cohort (Figure 2A); and 48.1 months (95% CI, 42.1–54.1 months), 24.3 months (95% CI, 19.4–29.2 months), and 9.7 months (95% CI, 5.1–14.3 months), respectively, in the validation cohort (Figure 2B). OS rates progressively decreased as the risk scores increased, differing significantly between low- and intermediate-risk groups and between intermediate- and high-risk groups in both cohorts (*p* ≤ 0.001; Figure 2).

**Figure 3:**
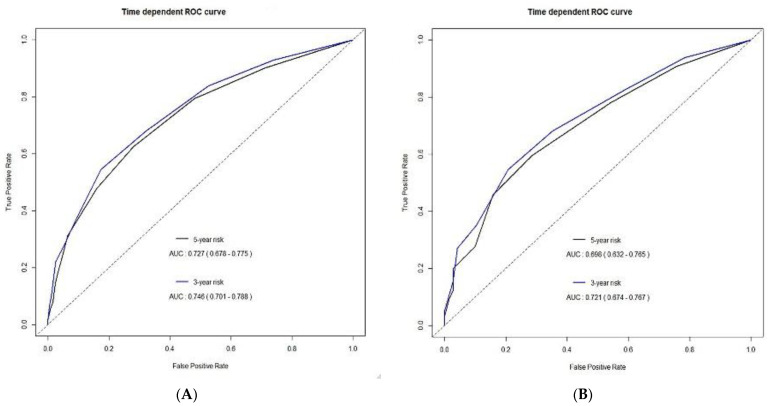
Time-dependent receiver operating characteristic (ROC) curves for overall survival in the (**A**) development and (**B**) validation cohorts.

Application of the predictive model to the validation cohort showed that the areas under the time-dependent ROC curves were 0.72 (95% CI, 0.67–0.77) and 0.70 (95% CI, 0.63–0.77) at 3 and 5 years, respectively. Figure 3 shows the time-dependent ROC curves for OS in the development and validation cohorts.

**Figure 4:**
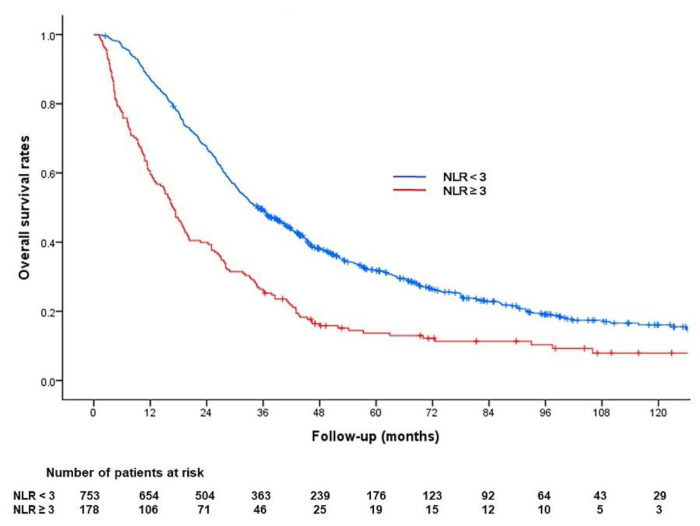
Kaplan–Meier analysis of overall survival according to the neutrophil/lymphocyte ratio (NLR) in the entire cohort. The median survival period was 34.9 months for patients with baseline NLR < 3 and 16.7 months for patients with baseline NLR ≥ 3 (*p* < 0.001).

The Kaplan–Meier curve determined with NLR in the entire cohort is shown in Figure 4.

### 3.3. Tumor Response 6 Months after TACE

Evaluation of tumor response at 6 months was not possible in 65 (7%) of the 931 patients because of mortality. Per protocol, these patients were classified as PD. Of the 931 patients, 392 (42%) achieved CR, 206 (22%) achieved PR, 37 (4%) showed SD, and 296 (32%) showed PD at 6 months. Multivariable logistic regression analysis showed that tumor diameter > 5 cm (odds ratio (OR), 2.18; 95% CI, 1.53–3.10; *p* < 0.001), tumor number ≥ 4 (OR, 3.44; 95% CI, 2.37–4.98; *p* < 0.001), infiltrative tumor type (OR, 3.18; 95% CI, 1.99–5.07; *p* < 0.001), AFP ≥ 200 ng/mL (OR, 1.76; 95% CI, 1.27–2.45; *p* = 0.001), and NLR ≥ 3 (OR, 3.35; 95% CI, 2.27–4.94; *p* < 0.001) were significant factors associated with PD 6 months after TACE (Table 3).

## 4. Discussion

The findings of the present study support the hypothesis that increased baseline NLR is a robust independent factor predicting OS after TACE for intermediate-stage HCC. This study found that NLR ≥3 was predictive of OS after TACE in the development cohort, with its predictive value clarified by cross-validation in the validation cohort. These findings, therefore, suggest that in addition to tumor burden, tumor biology, and underlying liver function, baseline patient immune status is an important factor predicting survival after TACE for intermediate-stage HCC.

Although several previous studies found that increased baseline NLR independently predicted outcomes after TACE in patients with unresectable HCC, the numbers of patients in previous studies, however, were relatively small and included a heterogeneous population consisting of patients with BCLC stages A, B, C, and D [22,23,24]. Thus, our results, using data from a large single-stage homogeneous cohort of 931 patients with BCLC stage B, in whom TACE is considered a standard of care, may firmly confirm the hypothesis about significant association of baseline NLR with OS after TACE in intermediate-stage HCC patients.

A recent, large-scale study developed a NLR-included prediction model for OS after TACE [25]. From their multivariate Cox’s regression analysis, tumor size, tumor number, AFP level, vascular invasion, Child–Pugh score, objective response after TACE, and NLR were selected as predictors of OS and incorporated into a 14-point risk prediction model (SNAVCORN) [25]. With cross validation, they showed that the prognostic performance of the SNAVCORN score including NLR in patients with HCC treated with TACE was remarkable [25]. However, their patient cohorts consisted of heterogenous population (BCLC A stage (*n* = 861), BCLC B stage (*n* = 598), BCLC C stage (*n* = 238)), and thus their prediction model may not be applicable to patients who underwent TACE for BCLC B stage HCC. To our knowledge, our study is first to introduce a new NLR-based model to predict OS after TACE for BCLC B HCC. In our study, tumor size, tumor number, tumor type, AFP level, Child–Pugh score, and NLR were incorporated into a 12-point risk prediction model. The combination of these six factors helped to identify three prognostic categories: low-, intermediate-, and high risk. We anticipate that our newly proposed NLR-based model may guide future treatment decisions, or subclassification for intermediate-stage HCC [26].

An optimal NLR cut-off value has not been determined to date. A study in which 145 patients with unresectable HCC were divided into two groups according to mean NLR found that the median OS after TACE was significantly lower in patients with high (≥3.3, *n* = 59) than normal (<3.3, *n* = 86) NLR (8 vs. 12 months, *p* = 0.001) [22]. In another study, in which the pre-TACE NLR cut-off value of five was chosen arbitrarily, median OS was significantly lower in the 18 patients with NLR > 5 than in the 86 patients with NLR ≤ 5 (4.2 vs. 14.9 months, *p* = 0.021) [23]. Furthermore, a study in 380 patients with unresectable HCC, in which patients were dichotomized by median pre-TACE NLR of 2.4, found that baseline NLR > 2.4 (HR, 1.34; 95% CI, 1.03–1.75; *p* = 0.027) was an independent prognostic predictor of poor OS after TACE [24]. A meta-analysis of more than 3000 patients with HCC, with threshold NLR values ranging from 1.9 to 5, found that NLR > 3 was a better predictor of OS than an NLR of 2–2.9 [27]. Based on this meta-analysis [27] and a previous systematic review [18], the present study chose an NLR cut-off value of three, finding that this cut-off value was a significant predictor of OS.

Radiologic responses of HCC 6 months after initial TACE or radioembolization were found to be predictive of OS [28], suggesting that radiologic response at 6 months may be useful in predicting OS or as a clinical trial end point [28]. In addition, the present study found that NLR ≥ 3 was a significant factor associated with PD 6 months after TACE. These findings are in agreement with those of a previous study [29], which found that increased baseline NLR (>3.5) was associated with PD as early as 2 months following initial TACE.

Our predictive model found that OS was significantly poorer in the high-risk group than in the low- and intermediate-risk groups. These findings suggest that TACE alone may be insufficient for these patients, and that other or additional therapeutic options should be considered. We found that baseline immune status (NLR) was a significant factor predicting OS and radiologic response 6 months after TACE. Thus, patients with high NLR before TACE may benefit from the addition of systemic treatments that can promote TACE-associated antitumor immune responses, thus achieving better outcomes [23]. The emergence of immunotherapy (e.g., immune check inhibitors) has rapidly expanded the treatment landscape for intermediate-stage HCC. Immune check inhibitors activate T lymphocytes to kill tumor cells by blocking the binding of PD-L1 to PD-1. TACE can increase tumor immunogenicity by stimulating a pro-immune inflammatory response and releasing tumor-associated antigens, which can increase systemic anticancer immune responses, including tumor-infiltrating cytotoxic CD8+ T cells [30], thus providing a solid rationale for the combination of chemotherapy with immunotherapy. Many ongoing trials (e.g., NCT03143270, NCT03572582, NCT04268888, NCT03397654, and NCT03099564) are investigating the efficacy of combinations of various immunotherapeutic agents (nivolumab or pembrolizumab) with TACE.

Because of the heterogeneity of intermediate-stage HCC, the outcomes of TACE in these patients also vary [31,32,33]. Attempts have been made to stratify these patients by subgroup, both for prognostic reasons and to develop optimal treatment strategies for each subgroup. In most previous studies, tumor burden (up-to-7 or up-to-11 criteria, 6-and-12 score) and underlying functional liver reserve (Child–Pugh score, ALBI grade) were used to subclassify patients with intermediate-stage tumors [17,26,34,35,36,37]. Serum AFP concentration has been incorporated into patient subclassification [17,37], but, to our knowledge, tumor type was not. Infiltrative HCC has been associated with poor prognosis [38]. Our multivariate analysis showed that infiltrative tumor type had the highest association (HR, 2.28; 95% CI, 1.73–3.01; *p* < 0.001) with poor OS. These findings indicate that infiltrative tumor type should be incorporated into the subclassification or pretreatment prediction model for intermediate-stage HCC.

This study had several limitations. First, it was a retrospective, single-center study, making it vulnerable to a variety of potential biases and limiting the generalizability of the results. However, we tried to minimize bias by cross-validation analysis of relatively large sample sizes. Second, cisplatin is not frequently used as the chemotherapeutic agent in TACE, and its use may have made the results difficult to generalize. Further external validation is needed to determine its reliability.

## 5. Conclusions

The current study supports the hypothesis that higher NLR is predictive of poor prognosis in patients who undergo TACE for intermediate-stage HCC. The pretreatment risk evaluation model developed in this study identifies important pretreatment risk factors. The combination of NLR and traditional tumor clinicopathological features may be used to establish treatment plans.

## Figures and Tables

**Table 1:** Baseline patient demographic and clinical characteristics.

Variable	All Patients	Development Cohort	Validation Cohort	*p*-Value
Patients	931	495	436	
Age, years	59.4 ± 9.8	58.2 ± 9.9	60.8 ± 9.6	<0.001
Sex				>0.999
Male	810 (87)	431 (87.1)	379 (86.9)	
Female	121 (13)	64 (12.9)	57 (13.1)	
Etiology				0.071
HBV	699 (75.1)	385 (77.8)	314 (72.1)	
HCV	101 (10.8)	52 (10.5)	49 (11.2)	
Others	131 (14.1)	58 (11.7)	73 (16.7)	
Child–Pugh class				0.556
A	812 (87.2)	435 (87.9)	377 (86.5)	
B	119 (12.8)	60 (12.1)	59 (13.5)	
Maximum tumor size, cm				0.792
≤5	424 (45.5)	223 (45.1)	201 (46.1)	
>5	507 (54.5)	272 (54.9)	235 (53.9)	
Number of tumors				0.262
2–3	421 (45.2)	215 (43.4)	206 (47.2)	
≥4	510 (54.8)	280 (56.6)	230 (52.8)	
Tumor involvement				0.893
Unilobar	373 (40.1)	197 (39.8)	176 (40.4)	
Bilobar	558 (59.9)	298 (60.2)	260 (59.6)	
AFP, ng/mL				0.254
<200	560 (60.2)	289 (58.4)	271 (62.2)	
≥200	371 (39.8)	206 (41.6)	165 (37.8)	
Neutrophil/lymphocyte ratio				0.867
<3	753 (80.9)	399 (80.6)	354 (81.2)	
≥3	178 (19.1)	96 (19.4)	82 (18.8)	

Data are shown as N (%) or mean ± SD. AFP, α-Fetoprotein; HBV, hepatitis B virus; HCV, hepatitis C virus.

**Table 2:** Results of univariable and multivariable Cox-proportional hazard models evaluating factors associated with overall survival after TACE in the development cohort.

Variable	Univariable Cox Regression Analysis				Multivariable Cox Regression Analysis						
	HR	95% CI		*p*-Value	Adjusted HR	95% CI		*p*-Value	β-Coefficients	Beta (W-Wref)/B	Risk Point
Maximum tumor size > 5 cm	1.73	1.43	2.09	<0.001	1.30	1.06	1.61	0.013	0.27	1.29	1
Tumor number ≥ 4	2.08	1.71	2.53	<0.001	1.67	1.35	2.05	<0.001	0.51	2.47	2
Infiltrative tumor type	3.11	2.40	4.02	<0.001	2.28	1.73	3.01	<0.001	0.82	3.99	4
Bilobar involvement	1.63	1.34	1.99	<0.001							
AFP ≥ 200 ng/mL	1.47	1.21	1.77	<0.001	1.23	1.01	1.50	0.042	0.21	1.00	1
Neutrophil/lymphocyte ratio ≥ 3	1.93	1.53	2.44	<0.001	1.41	1.10	1.81	0.007	0.35	1.67	2
Child–Pugh B	1.64	1.24	2.18	0.001	1.66	1.24	2.23	<0.001	0.51	2.46	2
Age	1.00	0.99	1.01	0.541							
Male sex	1.06	0.79	1.41	0.706							
Etiology				0.436							
HBV	1										
HCV	1.19	0.88	1.63	0.263							
Others	1.12	0.84	1.49	0.433							

AFP, α-Fetoprotein; HBV, hepatitis B virus; HCV, hepatitis C virus; HR; hazard ratio; CI, confidence interval.

**Table 3:** Results of univariable and multivariable logistic regression models evaluating factors predicting PD 6 months after TACE in the entire cohort.

Variable	Univariable Logistic Regression Analysis				Multivariable Logistic Regression Analysis			
	OR	95% CI		*p*-Value	Adjusted OR	95% CI		*p*-Value
Maximum tumor size > 5 cm	4.17	3.05	5.69	<0.001	2.18	1.53	3.10	<0.001
Tumor number ≥ 4	5.18	3.75	7.17	<0.001	3.44	2.37	4.98	<0.001
Infiltrative tumor type	6.06	4.02	9.13	<0.001	3.18	1.99	5.07	<0.001
Bilobar involvement	2.74	2.01	3.72	<0.001	1.38	0.96	2.00	0.085
AFP ≥ 200 ng/mL	2.52	1.89	3.34	<0.001	1.76	1.27	2.45	0.001
Neutrophil/lymphocyte ratio ≥ 3	4.36	3.10	6.14	<0.001	3.35	2.27	4.94	<0.001
Child Pugh B	1.67	1.13	2.47	0.011	1.40	0.87	2.24	0.166
Age	0.98	0.96	0.99	0.002	0.99	0.98	1.01	0.290
Male sex	1.37	0.92	2.04	0.116				
Etiology				0.859				
HBV	1							
HCV	1.03	0.66	1.61	0.900				
Others	0.89	0.59	1.35	0.608				

OR, odds ratio; CI, confidence interval; AFP, alpha fetoprotein; HBV, hepatitis B virus; HCV, hepatitis C virus.

## Data Availability

Data sharing not applicable.

## References

[1] Llovet J.M., Burroughs A., Bruix J. (2003). Hepatocellular carcinoma. Lancet.

[2] Llovet J.M., Bruix J. (2003). Systematic review of randomized trials for unresectable hepatocellular carcinoma: Chemoembolization improves survival. Hepatology.

[3] Tan C.K., Law N.M., Ng H.S., Machin D. (2003). Simple clinical prognostic model for hepatocellular carcinoma in developing countries and its validation. J. Clin. Oncol..

[4] Schoniger-Hekele M., Muller C., Kutilek M., Oesterreicher C., Ferenci P., Gangl A. (2001). Hepatocellular carcinoma in Central Europe:prognostic features and survival. Gut.

[5] Bruix J., Llovet J.M. (2002). Prognostic prediction and treatment strategy in hepatocellular carcinoma. Hepatology.

[6] Cabibbo G., Genco C., Di Marco V., Barbara M., Enea M., Parisi P., Brancatelli G., Romano P., Craxi A., Camma’ C. (2011). Predicting survival in patients with hepatocellular carcinoma treated by transarterial chemoembolisation. Aliment. Pharmacol. Ther..

[7] Jung M.R., Park Y.K., Jeong O., Seon J.W., Ryu S.Y., Kim D.Y., Kim Y.J. (2011). Elevated preoperative neutrophil to lymphocyte ratio predicts poor survival following resection of late stage gastric cancer. J. Surg. Oncol..

[8] Azab B., Bhatt V.R., Phookan J., Murukutla S., Kohn N., Terjanian T., Widmann W.D. (2012). Usefulness of the neutrophilto-lymphocyte ration in predicting short- and long-term mortality in breast cancer patients. Ann. Surg. Oncol..

[9] Li Y.-W., Qiu S.-J., Fan J., Zhou J., Gao Q., Xiao Y.-S., Xu Y.-F. (2011). Intratumoral neutrophils: A poor prognostic factor for hepatocellular carcinoma following resection. J. Hepatol..

[10] Fogar P., Sperti C., Basso D., Sanzari M.C., Greco E., Davoli C., Navaglia F., Zambon C.-F., Pasquali C., Venza E. (2006). Decreased total lymphocyte counts in pancreatic cancer: An index of adverse outcome. Pancreas.

[11] Mano Y., Shirabe K., Yamashita Y., Harimoto N., Tsujita E., Takeishi K., Aishima S., Ikegami T., Yoshizumi T., Yamanaka T. (2013). Preoperative neutrophil-to-lymphocyte ratio is a predictor of survival after hepatectomy for hepatocellular carcinoma: A retrospective analysis. Ann. Surg..

[12] Lué A., Serrano M.T., Bustamante F.J., Iñarrairaegui M., Arenas J.I., Testillano M., Lorente S., Gil C., De La Torre M., Gomez A. (2017). Neutrophil-to-lymphocyte ratio predicts survival in european patients with hepatocellular carcinoma administered sorafenib. Oncotarget.

[13] Weiyu X., Junyu L., Yi B., Yongchang Z. (2018). Prognostic role of neutrophil-to-lymphocyte ratio in unresectable hepatocellular cancer patients treated with trans-arterial chemoembolization. Transl. Cancer Res..

[14] European Association for The Study of The Liver (2018). EASL Clinical Practice Guidelines: Management of hepatocellular carcinoma. J. Hepatol..

[15] Gaba R.C., Lokken R.P., Hickey R.M., Lipnik A.J., Lewandowski R.J., Salem R., Brown D.B., Walker T.G., Silberzweig J.E., Baerlocher M.O. (2017). Quality improvement guidelines for transarterial chemoembolization and embolization of hepatic malignancy. J. Vasc. Interv. Radiol..

[16] Kim B.K., Shim J.H., Kim S.U., Park J.Y., Kim D.Y., Ahn S.H., Kim K.M., Lim Y.-S., Han K.-H., Lee H.C. (2016). Risk prediction for patients with hepatocellular carcinoma undergoing chemoembolization: Development of a prediction model. Liver Int..

[17] Lee I., Hung Y., Liu C., Lee R., Su C., Huo T., Li C., Chao Y., Lin H., Hou M. (2019). A new ALBI-based model to predict survival after transarterial chemoembolization for BCLC stage B hepatocellular carcinoma. Liver Int..

[18] Bannaga A., Arasaradnam R.P. (2020). Neutrophil to lymphocyte ratio and albumin bilirubin grade in hepatocellular carcinoma: A systematic review. World J. Gastroenterol..

[19] Lencioni R., Llovet J. (2010). Modified RECIST (mRECIST) assessment for hepatoeellular carcinoma. Semin. Liver Dis..

[20] Sullivan L.M., Massaro J.M., D’Agostino Sr R.B. (2004). Presentation of multivariate data for clinical use: The framingham study risk score functions. Stat. Med..

[21] Uno H., Cai T., Tian L., Wei L.J. (2007). Evaluating prediction rules for t-year survivors with censored regression models. J. Am. Stat. Assoc..

[22] Huang Z.-L., Luo J., Chen M.-S., Li J.-Q., Shi M. (2011). Blood neutrophil-to lymphocyte ratio predicts survival in patients with unresectable hepatocellular carcinoma undergoing transarterial chemoembolization. J. Vasc. Interv. Radiol..

[23] McNally M.E., Martinez A., Khabiri H., Guy G., Michaels A.J., Hanje J., Kirkpatrick R., Bloomston M., Schmidt C.R. (2013). Inflammatory markers are associated with outcome in patients with unresectable hepatocellular carcinoma undergoing transarterial chemoembolization. Ann. Surg. Oncol..

[24] Wang C., Wang M., Zhang X., Zhao S., Hu J., Han G., Liu L. (2020). The neutrophil-to-lymphocyte ratio is a predictive factor for the survival of patients with hepatocellular carcinoma undergoing transarterial chemoembolization. Ann. Transl. Med..

[25] Chon Y.E., Park H., Hyun H.K., Ha Y., Na Kim M., Kim B.K., Lee J.H., Kim S.U., Kim D.Y., Ahn S.H. (2019). Development of a new nomogram including neutrophil-to-lymphocyte ratio to predict survival in patients with hepatocellular carcinoma undergoing transarterial chemoembolization. Cancers.

[26] Bolondi L., Burroughs A., Dufour J.F., Galle P.R., Mazzaferro V., Piscaglia F., Raoul J.L., Sangro B. (2012). Heterogeneity of patients with intermediate (BCLC B) hepatocellular carcinoma: Proposal for a subclassification to facilitate treatment decisions. Semin. Liver Dis..

[27] Xiao W.-K., Chen D., Li S.-Q., Fu S.-J., Peng B.-G., Liang L.-J. (2014). Prognostic significance of neutrophil-lymphocyte ratio in hepatocellular carcinoma: A meta-analysis. BMC Cancer.

[28] Memon K., Kulik L., Lewandowski R.J., Wang E., Riaz A., Ryu R.K., Sato K.T., Marshall K., Gupta R., Nikolaidis P. (2011). Radiographic response to locoregional therapy in hepatocellular carcinoma predicts patient survival times. Gastroenterology.

[29] Cruz J.C., Watchmaker J.M., Albin M.M., Wang L., Wu G., Baker J.C., Fritsche M.R., Alexopoulos S.P., Matsuoka L., Fleming J.W. (2019). Neutrophil/Lymphocyte ratio predicts increased risk of immediate progressive disease following chemoembolization of hepatocellular carcinoma. J. Vasc. Interv. Radiol..

[30] Hickey R.M., Kulik L.M., Nimeiri H., Kalyan A., Kircher S., Desai K., Riaz A., Lewandowski R.J., Salem R. (2017). Immuno-oncology and its opportunities for interventional radiologists: Immune checkpoint inhibition and potential synergies with interventional oncology procedures. J. Vasc. Interv. Radiol..

[31] Prince D., Liu K., Xu W., Chen M., Sun J.-Y., Lu X.-J., Ji J. (2020). Management of patients with intermediate stage hepatocellular carcinoma. Ther. Adv. Med. Oncol..

[32] Yi P.S., Wang H., Li J.S. (2020). Evolution and current status of the subclassification of intermediate hepatocellular carcinoma. World J. Gastrointest. Surg..

[33] Golfieri R., Bargellini I., Spreafico C., Trevisani F. (2019). Patients with Barcelona Clinic Liver Cancer Stages B and C hepatocellular carcinoma: Time for a subclassification. Liver Cancer.

[34] Kim J.H., Shim J.H., Lee H.C., Sung K.-B., Ko H.-K., Ko G.-Y., Gwon D.I., Kim J.W., Lim Y.-S., Park S.H. (2017). New intermediate-stage subclassification for patients with hepatocellular carcinoma treated with transarterial chemoembolization. Liver Int..

[35] Wang Q., Xia D., Bai W., Wang E., Sun J., Huang M., Mu W., Yin G., Li H., Zhao H. (2019). Development of a prognostic score for recommended TACE candidates with hepatocellular carcinoma: A multicenter observational study. J. Hepatol..

[36] Hu K., Tang B., Yuan J., Lu S., Li M., Chen R., Zhang L., Ren Z., Yin X. (2019). A new substage classification strategy for Barcelona Clinic Liver Cancer stage B patients with hepatocellular carcinoma. J. Gastroenterol. Hepatol..

[37] Wang J.-H., Kee K.-M., Lin C.-Y., Hung C.-H., Chen C.-H., Lee C.-M., Lu S.-N. (2015). Validation and modification of a proposed substaging system for patients with intermediate hepatocellular carcinoma. J. Gastroenterol. Hepatol..

[38] Lee Y.J., Lee Y.R., Seo C.G., Goh H.G., Kim T.H., Yim S.Y., Han N.Y., Lee J.M., Choi H.S., Kim E.S. (2020). How should we assign large infiltrative hepatocellular carcinomas for staging?. Cancers.

